# “All about the money?” A qualitative interview study examining organizational- and system-level characteristics that promote or hinder shared decision-making in cancer care in the United States

**DOI:** 10.1186/s13012-020-01042-7

**Published:** 2020-09-21

**Authors:** Isabelle Scholl, Sarah Kobrin, Glyn Elwyn

**Affiliations:** 1 Dartmouth College, The Dartmouth Institute for Health Policy & Clinical Practice, Level 5, Williamson Translational Research Building, One Medical Center Drive, Lebanon, NH 03756 USA; 2 Department of Medical Psychology, University Medical Center Hamburg-Eppendorf, Martinistr. 52, W26, 20246 Hamburg, Germany; 3 Healthcare Delivery Research Program, National Cancer Institute, 9609 Medical Center Drive, Rockville, MD 20850 USA

**Keywords:** Shared decision-making, Implementation, Routine care, Cancer care, Oncology, United States, Organizational characteristics, Health system characteristics, Economic implementation barriers, Payment models, Financial incentives

## Abstract

**Background:**

Despite decades of ethical, empirical, and policy support, shared decision-making (SDM) has failed to become standard practice in US cancer care. Organizational and health system characteristics appear to contribute to the difficulties in implementing SDM in routine care. However, little is known about the relevance of the different characteristics in specific healthcare settings. The aim of the study was to explore how organizational and health system characteristics affect SDM implementation in US cancer care.

**Methods:**

We conducted semi-structured interviews with diverse cancer care stakeholders in the USA. Of the 36 invited, 30 (83%) participants consented to interview. We used conventional content analysis to analyze transcript content.

**Results:**

The dominant theme in the data obtained was that concerns regarding a lack of revenue generation, or indeed, the likely loss of revenue, were a major barrier preventing implementation of SDM. Many other factors were prominent as well, but the view that SDM might impair organizational or individual profit margins and reduce the income of some health professionals was widespread. On the organizational level, having leadership support for SDM and multidisciplinary teams were viewed as critical to implementation. On the health system level, views diverged on whether embedding tools into electronic health records (EHRs), making SDM a criterion for accreditation and certification, and enacting legislation could promote SDM implementation.

**Conclusion:**

Cancer care in the USA has currently limited room for SDM and is prone to paying lip service to the idea. Implementation efforts in US cancer care need to go further than interventions that target only the clinician-patient level. On a policy level, SDM could be included in alternative payment models. However, its implementation would need to be thoroughly assessed in order to prevent further misdirected incentivization through box ticking.

Contributions to the literature

- Organizational and health system characteristics appear to play a role in the implementation of shared decision-making (SDM). Little is known about the importance of those characteristics in cancer care in the United States (US).
- Interviews with diverse stakeholders revealed that financial interests of healthcare providers and organizations are a major barrier to SDM implementation in US cancer care.
- Leadership support and well-functioning multidisciplinary teams were seen as important to foster SDM implementation in US cancer care.
- All in all, the results show that SDM implementation in US cancer care will remain unlikely if economic barriers are not removed.

## Background

Despite decades of ethical [1], empirical [2–4], and policy support [5, 6], shared decision-making (SDM) has failed to become standard practice in US cancer care [7, 8]. Studies that investigated implementation difficulties have mainly described barriers at the patient and physician levels [9, 10], but few have considered how the structure of the US healthcare system itself may undermine adoption of SDM [11]. This is in line with the implementation science literature in general. While context factors have been recognized as potential influences on the uptake of innovations [12], they are still frequently neglected and empirical studies are limited [13].

SDM has been called the pinnacle of patient-centered care [14]. SDM is a communicative process where health professionals and patients aim to reach decisions by sharing the best available evidence, supporting the patient to consider options and achieve informed preferences [15]. SDM is especially relevant in cancer care. Treatment choice is often sensitive to individual preference, especially where options have different risks and benefits. However, as in other areas of healthcare, adoption of SDM in cancer care is slow [8, 16–18].

In a recent scoping review [11], we identified a range of organizational- and system-level characteristics that influence the implementation of SDM. Organizational characteristics that potentially influenced SDM implementation included issues of leadership, resources, workflows, and culture. Health system-level characteristics included aspects like health policies, incentives, education, and licensing. This review included studies conducted in a broad range of clinical settings in different countries and concluded that future work needs to assess the relevance of the identified characteristics in specific settings and healthcare systems in order to prioritize which characteristics need to be addressed and derive fitting strategies to do so.

Thus, the aim of our study was to better understand the role that the characteristics identified in the scoping review play in the cancer care setting in the USA.

## Methods

### Study design

We conducted a qualitative study using semi-structured telephone interviews, guided by criteria for reporting qualitative research (COREQ) [19]. The COREQ checklist can be found in Additional file 1.

### Setting and subjects

We interviewed stakeholders in US-based cancer care, e.g., researchers, clinicians, cancer center managers, representatives of government agencies, patient, and caregiver advocates.

### Sampling and recruitment

We adopted a purposive maximum-variation approach to sample participants that represented different ages, gender, specialty expertise (e.g., medical oncology, surgery, nursing, family medicine), SDM interest and expertise (e.g., ranging from not being familiar with the topic to vast expertise in SDM implementation), professional settings (e.g., HCPs working in fee-for-service environments vs. alternative payment models, community vs. academic cancer centers), and regions within the USA. We applied this sampling strategy with the core aim of identifying main shared themes covered by heterogeneous stakeholders in US cancer care. Potential participants were identified by our team’s networks at the Dartmouth Institute and the National Cancer Institute. At both institutions, IS approached key senior professionals with extensive knowledge of stakeholders active in US cancer care and asked them to provide ideas for potential interview partners for this study. This led to a successively growing list of potential participants from which the research team selected a heterogeneous set to invite to participate in the study. To attain theoretical data saturation, we planned 25 interviews by emailing invitations (and one reminder) to 36 stakeholders, assuming 60–70% response. There were no relationships established between the interviewer (IS) and the vast majority of the participants prior to study commencement. Participants were informed that IS conducted this study in her position as visiting scientist to the Dartmouth Institute.

### Data collection

One-time individual semi-structured telephone interviews were conducted at the workplace, recorded, and guided by a pre-tested interview schedule between January and April 2017 by IS, a female clinical psychologist with experience in the method. Each participant was asked to read a summary of our scoping review before their interview or given a synopsis. After a short introduction to the concept of shared decision-making and definitions of organizational and health system characteristics, participants were first asked to describe their impressions of the results of the scoping review. Then, they were asked to reflect on the relevance of the identified characteristics in their cancer-specific field of work and whether there were any other characteristics important for SDM implementation in cancer care not included in the review. Furthermore, the interviewer explored participants’ ideas for possible solutions to foster the implementation of SDM in cancer care. The full interview guide is displayed in Additional file 2. We collected age, gender and profession data. No field notes were made. Theoretical saturation was reached.

### Data analysis

We transcribed, anonymized, and imported the interviews into ATLAS.ti, a software for qualitative analysis. We conducted conventional content analysis [20] combining deductive and inductive coding, where one quarter of the interviews were initially coded by IS. Codes were reviewed and revised with GE (merging/rephrasing), followed by the development of a final code structure. Transcripts and data analysis were not returned to participants.

## Results

### Sample characteristics

Of the 36 invited, 30 (83%) participants consented to an interview; others did not reply or declined due to a lack of time. Of the 30, 13 were female and the sample had on average 24 years of work experience. They had diverse and often multiple roles: 10 identified as researchers, 9 as healthcare professionals, 7 as leaders of healthcare organizations, 4 as federal agency officers, 4 as patient/caregiver advocates, 2 as representatives of professional organizations, and 3 as having other roles (see details in Table 1). Interviews lasted 36 min on average (range 22–85).

**Table 1:** Characteristics of participants (*N* = 30)

Sex	
Female	13 (43%)
Male	17 (57%)
Age range (in years)	
25–34	1 (3%)
35–44	5 (17%)
45–54	7 (23%)
55–64	12 (40%)
65–74	5 (17%)
Work experience (in years)	
Mean (SD)	24 (9.8)
Range	9–43
Role(s) in cancer care^a^	
Researcher	10 (33%)
Healthcare professional (HCP)	9 (30%)
Healthcare organization leadership personnel^b^	7 (23%)
Federal agency officer	4 (13%)
Patient or caregiver advocate	4 (13%)
Representative of professional organization	2 (7%)
Other^c^	3 (10%)
Region within the USA	
South	12 (40%)
Northeast	8 (27%)
West	7 (23%)
Midwest	3 (10%)

^a^Answers were categorized based on participants’ replies to the question about their role. Multiple answers were possible. *N* = 7 reported to have two or more roles

^b^Includes leadership personnel of integrated health systems (*N* = 3)

^c^Includes *N* = 1 healthcare analyst, *N* = 1 oncology consultant, *N* = 1 health plan leadership personnel

### General appraisal of results from scoping review

Participants appraised the results of the scoping review [11] as *“comprehensive”* (ID#2, healthcare professional (HCP)) and as *“an accurate representation of what’s in the literature on how to implement SDM”* (ID#4, researcher). It was seen as relevant given that *“our government is embracing this [SDM]”* (ID#5, oncology consultant).

### Major theme: “All about the money”

The role of money and revenue generation was a dominant theme. Participants described financial aspects of cancer care delivery in the USA as strong barriers or disincentives to SDM implementation. A medical oncologist (ID#2) stated that *“the major incentives in the way our healthcare is structured (...) is economics. It’s revenue. It’s money.”*

A healthcare analyst (ID#1) questioned the very idea of implementing SDM in the USA: *“My core complaint (…) with cancer care particularly is that the industry has evolved to be all about the money. (…) What is the point of discussing the value of shared decision-making and your relationship with the patient when your intention is to do the wrong things to the patient in the first place? Because that is the intention of the American healthcare system, is to provide as much excess as possible, to make as much money as possible.” *A researcher added that for *“organizations (...), largely driven by revenue, there’s not necessarily a strong business case (...) for patient engagement, shared decision-making kinds of initiatives” *(ID#13).

#### Fee-for-service structure

The prevailing fee-for-service structure was criticized in two ways. Given that *“time is money”* (HCP, ID#2), the pressure to minimize visit lengths reduces motivation to compare treatment options with patients. As a federal agency officer (ID#16) stated: *“You’re paid more for (...) seeing more patients. That always puts time pressure on you (...) to spend less time with patients.” *Similarly, a CMO of a health plan (ID#29) expressed that* “right now [under the fee-for-service payment model] the amount of time involved to have the [SDM] discussion would economically disadvantage [HCPs].”* The fee-for-service model was also criticized for incentivizing treatment, which is more lucrative than not providing treatment: it also limits motivation to refer patients for alternative treatments. *“So if [providers] spend time (...) [doing shared decision-making] that actually results in a [patient’s] decision not to move forward, in the back of their mind, they’re impacting their income.”* (ID#24, leadership personnel). A federal agency officer (ID#16) admitted that *“there’s a financial incentive in fee-for-service to give chemotherapy.”*

Several participants mentioned the competing interests in community cancer care where providers buy cancer drugs themselves and bill the patient’s insurance company, generating a profit for themselves. As one leader in an academic cancer center, ID#21) said: *“In some places (...) I both prescribe you the chemotherapy in this room, and (...) [then] I sell it to you (…) down the hall.”* Even in academic contexts employed *“physicians (...) often have productivity incentives built into their compensation”* (leader of an integrated health system, ID#24).

Such quotes illustrate clearly that the idea of helping patients compare or defer treatment is simply not aligned with an institution’s financial interest.

#### Opaque treatment costs

Lack of transparency about out-of-pocket treatment costs was also discussed. High out-of-pocket costs cause financial burden. A patient and caregiver advocate (ID#7) emphasized that good decisions depend on being *“able to understand (…) the financial impact (…) of various treatment options, and on actual outcomes.”*

A cancer center leader (ID#6) commented that part of SDM has to be the *“financial burden that’s being put on the patient and their family in terms of (...) what’s the best course of therapy.”* However, as a caregiver advocate and provider (ID#18) said *“everything is so opaque (...) you still can’t find any of those numbers in real time.”* A patient advocate (ID#12) described his frustration when trying to find treatment costs: *“I eventually ended up talking to the insurance billing code people (…), and I found out that three different hospitals use significantly different billing codes for the exact same work.”* These examples illustrate how information opacity makes it very difficult, if not impossible, for patients to consider options on the basis of costs.

#### Direct-to-consumer advertising

A cancer program leader (ID#28) described the pharmaceutical industry’s direct-to-consumer advertising of cancer drugs as a barrier to SDM: *“Patients come in often knowing what [drug] they want based on industry-sponsored advertisements (...) and their advertising, their marketing, is substantially slicker and more powerful than my shared decision-making tool [laughs] or (…) ability. It’s hard to go against that.”*

#### Overcoming money- and revenue-related barriers

Interviewees had suggestions on how to overcome revenue-related barriers to SDM, such as introducing a billing code so that *“people get paid for doing shared decision-making”* (patient advocate, ID#12), or paying non-physicians to undertake SDM. The potential risks of incentivization were also raised. For example, a researcher (ID#13) said that extrinsic motivators could stimulate gaming, where: *“people end up sort of practicing to the metric.” *Others argued that if specialists only gets paid for certain treatments, they do not *“care (…) whether or not they get paid for the shared decision-making conversation” *(leading HCP and researcher in an academic cancer center, ID#21), indicating that incentivizing SDM conversations within the fee-for-service model will not be resolve the challenges to implementation.

Some participants thought that the introduction of value-based payment models might help. But a medical oncologist said (ID#2) that the devil would be in the details. If value was defined as the degree of SDM, *“then you’d have to figure out how (…) to measure [it].”* Bundled payment models were also mentioned as a possible solution. But in considering the Oncology Care Model, a federal agency officer (#ID16) said that “*you have to make the decision to receive chemotherapy to be part of the model,”* and therefore only rewarded one of many possible management decisions. Whether or not a purely salaried service, where clinicians *“receive no incentive (...) for [making] treatment decisions” *(leader of an integrated health system, ID#24) might be a possible solution was a matter of debate. One clinician felt such as system lacked *“incentives to work harder (...)”* (cancer center leader, ID#8).

### Other themes identified as important for SDM implementation

We identified agreement among participants that themes related to the roles of organizational leadership and multidisciplinary care coordination influenced SDM implementation.

#### Cancer center leadership

Participants emphasized the role of organizational hierarchy. A federal agency officer (ID#20) said that *“organizational leadership is one of the most important”* factors, echoed by a HCP and researcher (ID#27): *“It starts definitely at the top, so leadership is critical”* and by a patient and caregiver advocate (ID#12), who said that there has to be: *“support all the way up to the board of directors.”*

Participants said that adding SDM to vision and mission statements was *“not sufficient”* (patient advocate, ID#12). Leadership should operationalize actions, such as *“creating better pathways for implementing real SDM”* (federal agency officer, ID#22) and *“transparency”* (CMO of a health plan, ID#29) through feedback on performance, or even *“public shaming (...) that leads to more patient engagement” *(HCP and researcher, ID#15).

At the same time, some participants warned against organizational *“rhetoric” *(researcher, ID#13), e.g., mission and vision statements that are lip service and* “sound good”* (federal agency officer, ID#16), where SDM *“becomes a marketing term”* (patient and consumer advocate, ID#9). One researcher warned of *“hypocrisy”* (ID#13), of insufficient leadership to *“balance resources, time, energy, effort [required to do SDM] (...) against the expected economic [return]”* (leader of an integrated health system, ID#24). In other words, the less potential for revenue, the less leadership will provide tangible support for SDM.

#### Care coordination through teams, nurse navigators, and multidisciplinary clinics

Many participants emphasized the role of cancer care teams for SDM implementation. To quote: *“You know cancer is a team sport, (...) so having good communication skills not only with patients but also among the team members is important.”* (HCP and researcher, ID#15). Using additional disciplines such as social work, psychology, nursing navigators and coaches was viewed as a way to ensure SDM, given that patients do not always: *“feel comfortable sharing with their provider that they’re stuck in trying to decide what they want to do”* (HCP, ID#26), and *“[they] are less expensive than doctors” *(federal agency officer, ID#16). Some said that achieving SDM would need less hierarchical team structures, *“where the doctors are at the top, everybody else is at the bottom, and at the very, very bottom is the patient and their family” *(HCP, ID#2). Changing to a structure where *“the patient is really at the center and [where the members of] the healthcare team recognize their inter-dependency”* (HCP, ID#2) was suggested a step towards SDM implementation.

Finally, a few participants said that SDM in cancer care would be better facilitated by a multidisciplinary clinic approach, where patients meet with the relevant specialties and other services in a single clinic visit rather than by sequential referrals to specific specialists.

### Themes identified as controversial for SDM implementation

We also found disagreement about some themes regarding their importance for SDM implementation.

#### Electronic health records (EHRs)

The use of EHRs to prompt and document SDM processes or deliver decision aids led to different reactions. A federal agency officer (ID#16) thought that “*having (...) checkboxes that you did shared decision-making [is] not terribly effective”* given a lack of verification. Others questioned feasibility: *“it’s going to be three years before the EHR programmers can implement a DA [decision aid] in the system” *(patient advocate, ID#12). Others felt that the integration of tools into EHR could be *“huge, because that’s the future”* (oncology consultant, ID#5). Some speculated about the potential for algorithms that *“offer more precise [risk] estimates for (...) the patient”* (federal agency officer, ID#22) compared to decision aids based on population averages, or envisioned a *“tech[nology] platform that includes the EHR (...) [as well as] a dashboard for both the patient and the clinical side”* (ID#7, patient and caregiver advocate) where *“[patients] have a place where you can put down (...) preferences”* (representative of professional organization, ID#25).

#### Accreditation and certification

Fostering SDM implementation by making it a requirement for organizational accreditation and certification was suggested by some participants. One researcher (ID#17) expressed that SDM implementation becomes a requirement for every *“NCCN [National Comprehensive Cancer Network] (...) or an NCI [National Cancer Institute] designated cancer center (...).” *Others disagreed: *“we are over accredited and over-certified”* (cancer center leader, ID#6), echoing the view of a federal agency officer (#ID16) who said that: *“people probably will just find a way to say that they did it and not really do it”* given current measurement limitations.

#### Legislation

Participants had strong opposing reactions to the idea of mandates for SDM by legislation at either state or federal level: *“It’s a step in the right direction”* said a healthcare analyst (ID#1). Two cancer center leaders rejected the idea because *“the more government becomes involved the more complex they make it* “ (ID#6), and therefore *“the higher the likelihood [of] (...) physician pushback”* (ID#19).

## Discussion

### Summary and reflection of findings

The dominant theme in the study data was that concerns about the absence of revenue, or indeed, the likely loss of revenue, were a major barrier preventing the implementation of SDM. Many other factors were prominent as well, but the view that SDM might impair organizational or individual profit margins and reduce the income of some health professionals was widespread. While the study focused on organizational- and system-level characteristics that promote or hinder SDM in US cancer care, this major theme also touches the individual clinician-patient level, as individual clinicians’ actions related to revenue generation were also discussed. However, they should not be villainized as greedy professionals, but seen as responding to the system in which they work.

On the organizational level, having leadership support for SDM within cancer care delivery organizations and multidisciplinary teams were viewed as critical contributors to potential future implementation. On the health system level, views diverged on whether embedding tools into EHRs, making SDM a criterion for accreditation and certification, and enacting legislation could promote the implementation of SDM.

### Strengths and limitations

We do not claim, given the sample, that this qualitative study provides strong generalizable data. However, the purposive maximum variation strategy does provide a range of stakeholder views about the place of SDM in US cancer care. It is an inherent feature of this sampling approach that the selection of participants with diverse characteristics relies on the research team’s judgment. To minimize this effect, we consulted with key experts in our networks to identify potential participants. Yet, the selection might have been biased. Nevertheless, the identified themes are significant because they emerged out of this heterogeneous sample. At the same time, the chosen approach does not allow an investigation of differences between subgroups of participants, which has to be considered as a further limitation of this study. Of the purposively invited sample of stakeholders, 83% agreed to participate in an interview. In order not to compromise the anonymity of the participants, we did not gather extensive information on demographic and professional characteristics. It can be seen as a further limitation. However, this also ensured the necessary confidentiality for gathering sensitive data about how organizational- and system-level factors influence cancer care in the USA.

### Comparison to previous work

Most previous studies that have examined the implementation of SDM have not reported the dominant role of revenue generation [11, 21]. The US healthcare system may be particularly sensitive to impact on revenue generation. The US system differs from most OECD countries, being predominantly based on a fee-for-service structure, having low levels of purely salaried physicians [22], and where a high percentage of GDP is spent on healthcare [23]. Cancer specialists in the USA earn much more than in other countries [24], and US oncologists charge more for office-administered drugs than other specialists [25]. However, the impact on revenue may also apply in other contexts, as found in a Dutch study that describes financial interests of HCPs as one of many barriers towards SDM implementation in multidisciplinary sciatica care [26]. New evidence that payment structures influence SDM implementation comes from an example in the Netherlands, where better and more patient-centered care at lower costs was achieved by moving away from fee-for-service payment model, and by changing hospital structures and culture [27].

Furthermore, the rising costs of cancer treatment, although a challenge in many countries, have been described as particularly problematic in the USA [28, 29]. The combination of a comparatively high proportion of people without health coverage [30] and rising out-of-pocket costs paid by insured patients with cancer [31] lead to substantial financial toxicity in patients with cancer [32]. This burden can be aggravated by the opacity of treatment costs, which was described in our study and which is uniquely relevant in the US health care system [33]. Despite the challenges in identifying overall costs, out-of-pocket costs need to be addressed early on in treatment decision-making processes [34], as they have been shown to influence patient preferences and treatment adherence [35]. Two recent studies have shown that SDM tools can trigger cost discussions, but that more support is needed to help HCPs address treatment cost and financial distress adequately [36, 37].

System-level changes, such as legislative mandates and accreditation requirements, were viewed with some skepticism, comparable to HCPs’ attitudes towards accreditation in general [38]. At the same time, legal requirements for quality improvement strategies and adoption of accreditation programs are known drivers of change [39, 40]. In fact, even in the USA, several healthcare and cancer-specific policies have recommended the implementation of SDM [5, 41]. The US National Comprehensive Cancer Network (NCCN) support the use of SDM in their patient guidelines, e.g., [42, 43], and the communication consensus guideline by the American Society of Clinical Oncology includes SDM [44]. Nevertheless, these policies have had little impact, and no enforcement. In relation to reducing financial toxicity, it has been pointed out that health policy (e.g., price negotiations, value-aligned pricing strategies) and regulatory interventions (e.g., revision of drug approval regulations) could play a crucial role in eliminating low-value care, i.e., the use of expensive treatment options with minimal to no clinical benefits [33]. Another suggested strategy highlights the role of cancer societies, which could develop cost-conscious clinical practice guidelines [34]. Such changes could indirectly foster SDM, by mitigating the necessity of revenue generation as a barrier to SDM implementation.

A number of studies point to the important roles of organizational leadership and care coordination through multidisciplinary teams [11], and our findings echo results of a study that found the quality of healthcare can be improved by supportive and visionary leadership [45]. Several participants reported organizational leaders having only lip service commitment to SDM, illustrating the known phenomenon where patient-centered organizational statements represent rhetoric more than real commitment [46].

Our results can also be compared to a multi-study analysis investigating attributes of context found relevant by HCPs in the implementation of evidence-based practices [13]. The main theme of our study was also found as one of several core contextual factors in this secondary analysis of 145 interviews from 11 studies in different clinical contexts (not including oncology) [13]. That study also revealed that regulatory and legislative standards were less commonly described attributes that varied in their relevance across clinical contexts [13].

### Implications for future research

Future SDM implementation research would benefit from comparing the impact of a multicomponent SDM implementation program located in different payment models. As recently pointed out by Roberts and colleagues [47], the use of health economic evaluations in implementation science is limited. An economic evaluation of SDM implementation, possibly using a mixed methods design [48], would shed light on this under researched area. Also, future SDM implementation efforts in US cancer care need to go further than interventions targeting only the clinician-patient level (e.g., training for individual HCPs). Our study generally highlights the need to further investigate outer setting variables in implementation studies. In order to do so, it is necessary to develop further tools to measure those variables [13] and to distinguish between modifiable and unmodifiable variables [49]. Last, but not least, future research on treatment decision making processes should further investigate the role of treatment costs and financial toxicity.

### Implications for health care organizations and health policy

On an organizational level, efforts that combine SDM implementation with other quality improvement initiatives could be more effective [50]. On a policy level, SDM could be included in bundled payment formats [51] or become a mandatory requirement for reimbursement within the fee-for-service structure and enforced. The challenge would be the need to accomplish fair and rigorous assessment in order to prevent further misdirected incentivization or gaming.

## Conclusions

All in all, the results show it is unlikely that SDM will become implemented in US cancer care without structural and policy changes that tackle the current economic barriers towards the approach.

## Supplementary information

**Additional file 1.** Consolidated criteria for reporting qualitative studies (COREQ): 32-item checklist

**Additional file 2.** Interview guide

## Data Availability

The data that support the findings of this study are available on reasonable request from the corresponding author (IS). Signing a data use/sharing agreement will be necessary, and data security regulations both in the United States and in the country of the investigator who proposes to use the data must be complied with. The data are not publicly available due to them containing information that could compromise research participant privacy/consent.

## Abbreviations

CMO: Chief Medical Officer
COREQ: Consolidated criteria for reporting qualitative research
DA: Decision Aid
EHR: Electronic health record
GDP: Gross domestic product
GE: Glyn Elwyn
HCP: Healthcare professional
IS: Isabelle Scholl
ID#1 –29: Identification numbers participants
NCCN: National Comprehensive Cancer Network
NCI: National Cancer Institute
OECD: Organization for Economic Cooperation and Development
SDM: Shared decision-making
