# Supplementary material for: “All about the money?” A qualitative interview study examining organizational- and system-level characteristics that promote or hinder shared decision-making in cancer care in the United States
Source: Implement Sci. 2020 Sep 21;15:81. doi: 10.1186/s13012-020-01042-7 (PMC7507661; doi:10.1186/s13012-020-01042-7)
Supplement: Supplementary file 2 — Additional file 2. Interview guide [file 13012_2020_1042_MOESM2_ESM.docx]

**Additional file 1. Interview guide**

*[Before the telephone interview takes place, the interviewee will receive a written summary of the results of the scoping review.]*

**ESTABLISH TRUST**

**LISTEN INTENTLY**

**BE CURIOUS AND (APPROPRIATELY) INQUISITIVE**

**PURSUE UNANTICIPATED DIRECTIONS**

***As stated in the Interview Information Sheet that I sent you, this interview will be audio-recorded, then transcribed and anonymized for analysis. Did you read the Information Sheet? Do you agree to this procedure?***

*As you know, I am interested in understanding organizational- and system-level factors that influence the implementation of shared decision-making in routine cancer care. In this interview today I would like to learn more about your perspective on this topic. There are no right or wrong answers; I am interested in your experience.*

*Before we start, I would like to briefly define what I mean by shared decision-making, and by organizational and system levels of care to make sure we are on the same page.*

*With shared decision-making, I mean a collaborative process that allows patients and their providers to make health care decisions together. Patients are supported to consider options by taking into account the best clinical evidence available, as well as the patient’s values and preferences. Organizational-level factors are characteristics of a healthcare organization or institution (for example a hospital, a cancer center, a practice) that might affect the implementation of shared decision making; they are sometimes also described as institutional-level factors or factors on the meso level of care. This definition takes into account that several sublevels can be nested within an organization, e.g. factors related to a whole hospital, to a certain department within a hospital, to a certain team in a department, etc. System-level factors are characteristics of the health care system, meaning that they are factors that influence different organizations. Examples could be policies, reimbursement schemes, certification standards. Sometimes they are also described as factors on the macro level of care.*

*I would like to start with your thoughts on the results of the scoping review we conducted.*

*Did you have a chance to look at it?*

→ if interviewee answers “yes” - ask if he/she has document in front of him/her and continue with first question

→ if interviewee answers “no” - ask if he/she has document in front of him/her, briefly walk him/her through results and continue with first question

| **PART ONE: Barriers and facilitators**  *Let’s start with the organizational- and system-level characteristics identified in the review.* | |
| --- | --- |
| 1 | As [a researcher/a health care provider/a patient advocate/ someone working in health policy/etc *- be as precise as possible*], what are your first impressions on the results of the review? |
| 2 | What are your own experiences with the identified organizational- and system-level factors [at your place of work/in your center/etc]?   1. Which do you consider most important? 2. Which do you consider least important? |
| 3 | Are there any other organizational-level or system-level factors (or barriers and facilitators) that you consider important in regards to the implementation of shared decision making in cancer care; I mean any that were not identified in the review? |

| **PART TWO: Solutions**  *I would now like to look at possible solutions to foster the implementation of shared decision-making in cancer care.* | |
| --- | --- |
| 5 | What are your first thoughts on the organizational- and system-level solutions identified in the review? |
| 6 | What are your own experiences [at your place of work/in your center/etc] with implementing shared decision-making? Which organizational- or system-level solutions were helpful or could be helpful? |
| 7 | Do you have any other ideas to address the characteristics found in the review in order to foster the implementation of shared decision making in cancer care? |

| **PART THREE: Demographic questions**  *As a last part of the interview, I would like to you a couple of demographic questions.* | |
| --- | --- |
| 10 | What is your professional background? |
| 11 | What is your current role (official title)? |
| 12 | How long have you been working in cancer care / research / health policy / as a patient advocate / etc? |
| 13 | In what age bracket are you?  18-24, 25-34, 35-44, 45-54, 55-64, 65-74, above 75 |
| 14 | What is your gender? |

***THANK YOU*** *for your participation and your valuable input into this research project!*

***General probes:***

*[Silent probe] Make enough pauses (Cave: risk of misunderstanding in telephone interview)*

*[Neutral probe] Encouraging “I see” or “uh-huh”*

*[Direct probe] Can you tell me more about that?*

*[Echo probe] Repeat last statement and ask interviewee to continue (CAVE: risk of suggestive question)*

*[Clarifying probe]What did you mean when you said “___________”?*

*[Clarifying probe] You just referred to “____________.” What did you mean by _________”*

*[Detail probe]Who? Where? What? When? How?*
